# Studies of Multiferroic Palladium Perovskites

**DOI:** 10.1038/s41598-018-38411-8

**Published:** 2019-02-08

**Authors:** Dhiren K. Pradhan, Ajay K. Mishra, Shalini Kumari, Abhisek Basu, Maddury Somayazulu, Elzbieta Gradauskaite, Rebecca M. Smith, Jonathan Gardner, P. W. Turner, Alpha T. N’Diaye, M. B. Holcomb, Ram S. Katiyar, Peng Zhou, Gopalan Srinivasan, J. M. Gregg, J. F. Scott

**Affiliations:** 1 Geophysical Laboratory, Carnegie Institution for Science, Washington, DC 20015 USA; 2 Department of Physics and Institute for Functional Nanomaterials, University of Puerto Rico, San Juan, PR 00931 USA; 3 Department of Physics and Astronomy, West Virginia University, Morgantown, WV 26506 USA; 4 Present Address: Department of Earth, Ocean & Atmospheric Sciences, Florida State University, Tallahassee, Florida 32306 USA; 5 Department of Chemistry and Department of Physics, University of St. Andrews, St, Andrews, KY16 ST UK; 6 Present Address: Department of Materials, ETH Zurich, Vladimir-Prelog-Weg 4, 8093 Zurich, Honggerberg, 8093 Zurich Switzerland; 7 Centre for Nanostructured Media, School of Maths and Physics, Queen’s University of Belfast, Belfast, BT7 1NN Northern Ireland UK; 8 NCEM, Molecular Foundry, Lawrence Berkeley National Laboratory, Berkeley, California 94720 USA; 9 Department of Physics, Oakland University, Rochester, MI 48309-4479 USA

## Abstract

We have studied the atomic force microscopy (AFM), X-ray Bragg reflections, X-ray absorption spectra (XAS) of the Pd L-edge, Scanning electron microscopey (SEM) and Raman spectra, and direct magnetoelectric tensor of Pd-substituted lead titanate and lead zirconate-titanate. A primary aim is to determine the percentage of Pd^+4^ and Pd^+2^ substitutional at the Ti-sites (we find that it is almost fully substitutional). The atomic force microscopy data uniquely reveal a surprise: both threefold vertical (polarized out-of-plane) and fourfold in-plane domain vertices. This is discussed in terms of the general rules for Voronoi patterns (Dirichlet tessellations) in two and three dimensions. At high pressures Raman soft modes are observed, as in pure lead titanate, and X-ray diffraction (XRD) indicates a nearly second-order displacive phase transition. However, two or three transitions are involved: First, there are anomalies in c/a ratio and Raman spectra at low pressures (P = 1 − 2 GPa); and second, the c/a ratio reaches unity at ca. P = 10 GPa, where a monoclinic (M_c_) but metrically cubic transition occurs from the ambient tetragonal P4 mm structure in pure PbTiO_3_; whereas the Raman lines (forbidden in the cubic phase) remain until ca. 17 GPa, where a monoclinic-cubic transition is known in lead titanate.

## Introduction

Palladium is an unusual element for multiferroic materials because it is often nonmagnetic. Under normal conditions as a component of crystals it has a borderline magnetic instability. Consequently it has been studied less than transition metals such as Fe, Co, Ni, or Mn as a component in multiferroelectric compounds. Recently it has been reported that up to 30% Pd-substitution is possible in PbTiO_3_ (PTO) and in PbZr_(1-x)_Ti_x_O_3_ (PZT) with resulting magnetoelectric behaviour at room temperature. However, the initial reports did not fully characterize the materials to the degree required for device application, and in particular did not quantify how much Pd is exsolved to the grain surfaces (ideal for catalysis) or substitutional (ideal for magnetoelectrics), nor what percentage is at the A- and B-sites of the perovskite structure. The present study reveals unexpectedly that most of the Pd is in Pd + 4 in both PTO and PZT.

The host material in this report, lead titanate, has been of considerable interest as a ferroelectric material for more than fifty years, stimulated in part by the Raman and infrared studies of its soft mode by Burns and Scott^1,2^ and others^3,4^.

More recently great interest in the effects of hydrostatic pressure^5–9^ and uniaxial stress, especially “negative” stress produced by chemical substitution^10^, has arisen. ref.^10^ reveals an amazing polarization of 236 μC/cm^2^ without any applied stress (merely from interfacial strain), almost twice that in any other known material; this makes it attractive in the near future for Pd-substitution to try to achieve a super-multiferroelectric room-temperature magnetoelectric.

Unfortunately, the hydrostatic pressure studies do not agree with each other for the (P, T) phase diagram of PbTiO_3_, particularly with regard to the presence of one or two monoclinic phases (M_c_ and M_a_) near P = 10 GPa. This can be due to slightly non-hydrostatic pressure or to local heating under pressure. Of interest in the present context is the substitution of palladium or other magnetic ions into lead titanate^11,12^. Palladium is sometimes magnetic, and this affords the possibility of a room-temperature multiferroic. Pd in PbTiO_3_ is expected to go into the B-site, replacing Ti^+4^, where it is an exact fit to ionic size (Pd^+4^ is 0.615 Å; Ti^+4^ is 0.605 Å)^13,14^ and valence. However, indirect evidence shows that much of it goes into the Pb A-site, which is nominally an exact match for (6-coordinated octahedral site) Pd^+2^ at 0.86 Å radius^12–14^. In fact, magnetism in Pd is usually produced or enhanced by stress or electric fields, so Pd in Pb sites may facilitate that. It is important to note that the introduction of both Pd and Pt substitutional in perovskite oxides has recently been successful for catalysis^15–17^.

## Experimental Methods

10 and 30% Pd-substituted PbTiO3 Samples were prepared using a standard solid-state synthesis method. Stoichiometric amounts of precursor powders (PbO, TiO2, and PdO) were manually compacted and calcined with a PbTiO3 sacrificial powder. Powders were calcined at 873 and 1073 K for 1–10 hours, then sintered at 973–1173 K for 4–16 hours to minimize the formation of Pd-containing side products. The detailed synthesis conditions of Pd substituted PbZr_(1-x)_Ti_(x)_O_3_ PZT ceramics are reported elsewhere^13^. Field emission scanning electron microscopy (FESEM) images were captured with help of a Zeiss Auriga field emission SEM equipped with an Oxford Instruments X-Max 80 (SDD) Energy dispersive spectroscopy (EDS) system operated with an accelerating voltage of 15–30 kV to study the surface morphology and elemental analysis. For high-pressure synchrotron studies, samples of Pd-substituted PbTiO3 were loaded into diamond anvil cells equipped with gem- quality diamonds with a culet size of 300 µm. The sample chamber was obtained by drilling a 150-µm hole in a tungsten gasket pre-indented to 20 GPa with a corresponding thickness of 50 µm. Neon was used a pressure medium and ruby as pressure standard. Pressure was measured using the ruby fluorescence and the non-hydrostatic ruby gauge. X-ray diffraction data was collected at the synchrotron beam-line 16-ID-B at HPCAT of APS. The 200 µm incident x-ray beam at 0.4066 Å was focused using Kirk-Patrick Baez mirrors down to 10 µm and cleaned up using a tantalum pinhole. 2D diffraction data was recorded on a PILATUS detector with a spatial resolution of 172 µm at a distance of 205 mm from the diamond anvil cell. The limited opening angle of the diamond cell allowed data to be collected over a two-theta range of 5–25° corresponding to 4.5-0.9 Å in d-spacing. The 2-dimensional diffraction images were integrated into a diffraction patterns using DIOPTAS. The detector was calibrated using a CeO2 standard. High pressure Raman spectroscopy study was performed in a Boehler plate diamond anvil cells using 300 µm culet ultra-low fluorescence diamonds. A stainless steel gasket was pre-indented to 40 µm and 100 µm sample hole was made using an infra-red laser driller. The sample was loaded along with few ruby grains (pressure standard) in argon pressure transmitting medium. The error in pressure was ±0.1 GPa. All the Raman spectrum was collected using a 532 nm laser and 1800 lines/mm grating. XAS measurements were taken using the beamline 6.3.1 Magnetic Spectroscopy/Materials Science at Advanced Light Source (ALS) of Lawrence Berkeley National Laboratory, Berkeley, USA.

For magnetoelectric measurements the samples were polished and electrodes of Ti-Pt (40 nm/600 nm) were deposited by RF sputtering. They were then poled in an electric field by heating to 400 K and then cooling back to room temperature in an electric field E = 5 kV/cm. The sample with 30% Pd, however, had a much higher leakage current than the sample with 10% Pd and poling had to be done with E = 1 kV/cm. For direct-ME measurements, i.e., sample response to an applied magnetic field, samples were placed in an aluminum box in order to reduce to any noise voltage and then subjected to a bias magnetic field H produced by an electromagnet an ac magnetic field H_ac_ = 1 Oe at 100 Hz produced by a pair of Helmholtz coils. The voltage induced V across the sample thickness (t) was measured with a lock-in-amplifier. The ME voltage coefficient MEVC = V/(H_ac_ t) was measured as a function of H for two different field orientations: (i) H and H_ac_ parallel to each other and parallel to the sample plane and (ii) Both fields perpendicular to the sample plane.

## Results and Discussion

### Scanning Electron Microscopy

There are some preliminary concerns to satisfy first: It has not previously been established with certainty that these samples involve complete substitution of Pd^+2^ and Pd^+4^-ions in the PbTiO_3_ lattice, especially at A-sites; it had been considered possible that some of the palladium is exsolved to the surfaces^15–19^ although that would not be magnetic and hence the measured magnetism implies such a percentage is not large. Parenthetically we note that this would not necessarily be a bad outcome, since it might favor use for catalysis. It is also possible that despite the direct measurement of magnetoelectric constants in Pb(Zr_0.20_Ti_0.80_)_0.70_Pd_0.30_O_3-δ_^13^ some of the magnetism comes from PdO or PbPd_3_, which are magnetic metals with centered, non-ferroelectric structures. However, as shown below, the present samples have almost fully substitutional Pd.

**Figure 1:**
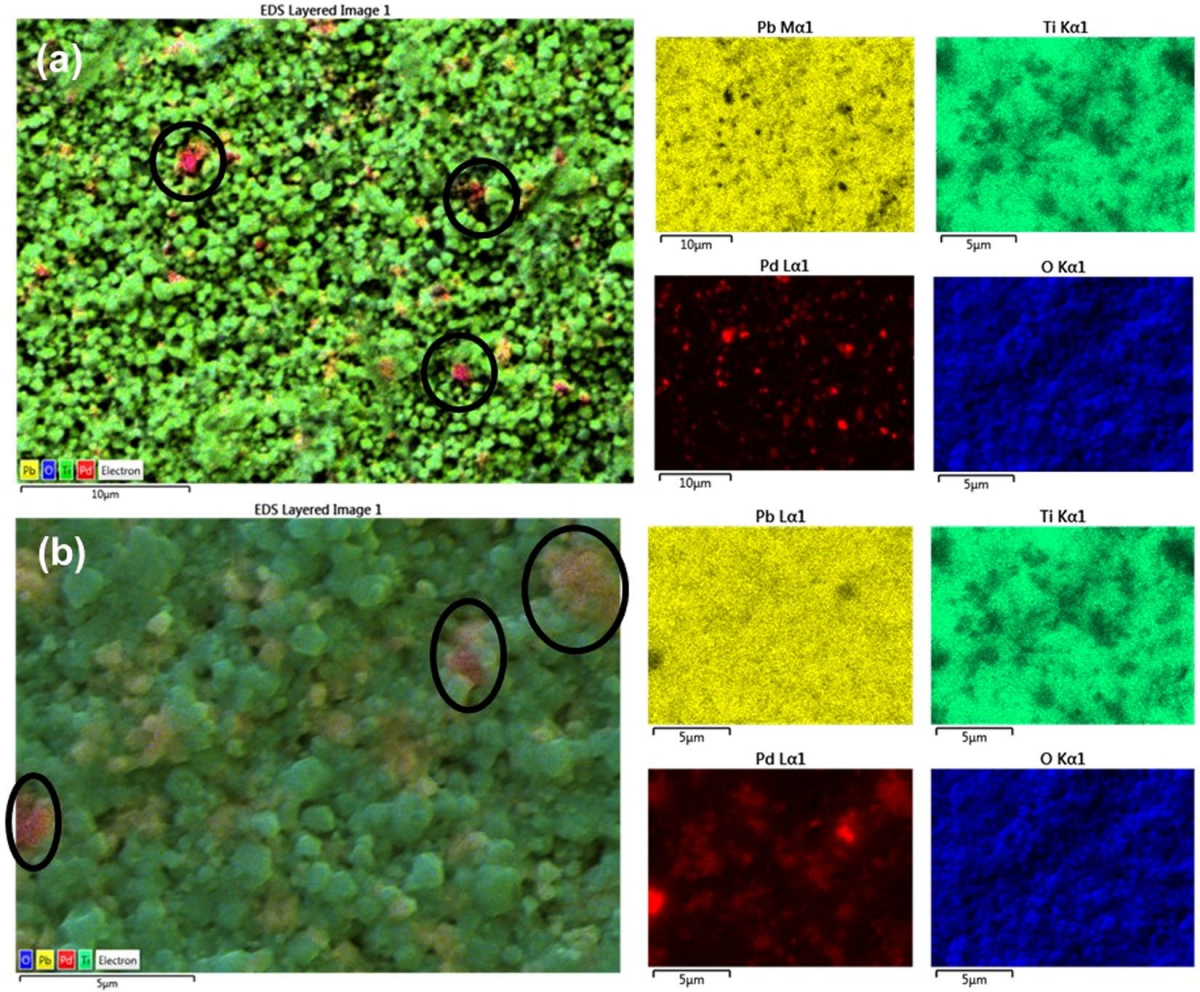
Elemental mapping of (**a**) Pb(Ti_0.90_Pd_0.10_)O_3-δ_, (**b**) Pb (Ti_0.70_Pd_0.30_)O_3-δ_ respectively.

**Figure 2:**
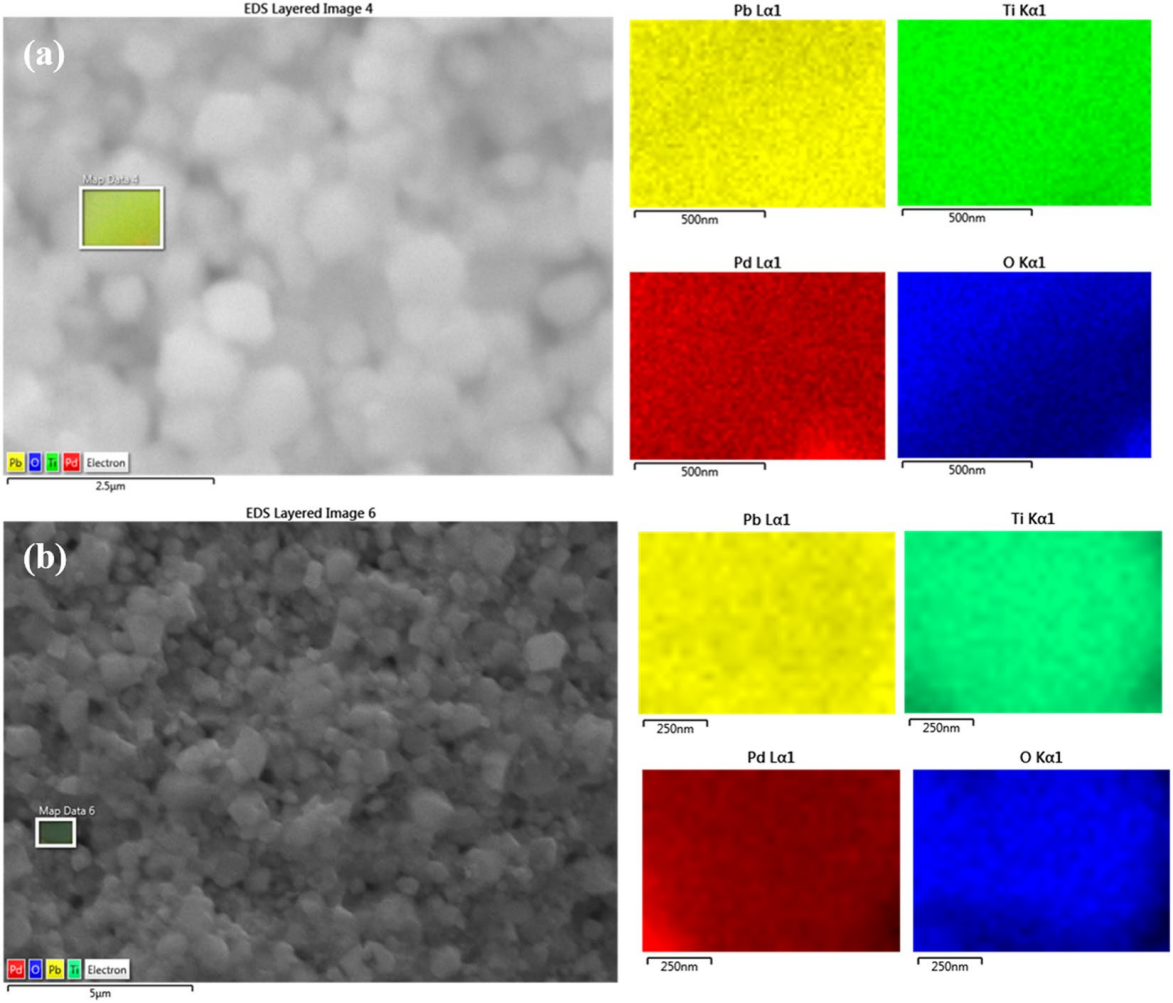
Elemental mapping in a single grain (**a**) Pb(Ti_0.90_Pd_0.10_)O_3-δ_, (**b**) Pb(Ti_0.70_Pd_0.30_)O_3-δ_.

To determine this directly, we employed SEM, together with EDS, with the results shown in Figs 1 and 2. Previous work on Pd substitutional in perovskite oxides has emphasized Pd^+3^ at the B-sites (with nearby oxygen vacancies to convert Pd^+4^ to Pd^+3^), replacing Fe or Co^18^. In ref.^18^ Pd incorporation was achieved for La(Fe,Pd)O_3±δ_ and La(Co,Pd)O_3±δ_ that showed the XANES line of Pd to be in octahedral coordination. Up to 10% Pd^+4^ has been reported substitutional into the B-site of other perovskite oxides with complete stability^19–21^, in another case, 20%^22^, and 100% Pd^+2^ in Sr_2_PdO_3_ with excellent medical applications^23,24^, >8% Pd can be substituted in rutile TiO_2_ (however, 23% produces amorphous samples)^25^.

In 10% and 30% Pd substituted PTO samples, Pd is uniformly distributed in a large area except few sites (black circled areas) and Pd is uniformly distributed even in a single grain (Fig. 1). This is important, because it establishes unambiguously that the Pd is substitutional. In order to check the presence of all elements, we have carried out elemental mapping on the pellet of 10% Pd-substituted PTO. The color images illustrate quantitative analyses of each elements Pb (yellow), Ti(green), Pd (red) and O(blue) present in the system. From Fig. 1(a) it is clearly seen that all the elements are homogeneously distributed in a large area of around 30 × 25 μm^2^. It is also observed that, Pd (red colored) is distributed homogeneously throughout the 10% Pd substituted PbTiO_3_ sample except some area (black circles). The distribution of Pd is also studied in a single grain of 10% Pd substituted PbTiO_3_ sample and we found that in a single grain also it is distributed homogeneously (Fig. 2(a)). These data are sufficient to establish that PbTiO_3_:Pd is a room-temperature multiferroic and that the ferroelectricity and ferromagnetism do not arise from separate phases or separate grains in the ceramic.

In 30% Pd-substituted PTO samples, Pd is not uniformly distributed in a large area it forms clustering in few sites see black circle area of Pb_2_PdO_4_ in Fig. 1(b). But Pd is uniformly distributed even in a single grain (Fig. 2(b)). Regarding the % of Pd in PbTiO_3_:Pd, this is given below in Tables 1 and 2.

**Table 1:** Observed % Pd in nominal 10% Pd-substituted PTO sample.

10% Pd substituted PbTiO_3_ Pb(Ti_0.9_Pd_0.1_)O_2.9_	Calculated wt%	Observed wt % from SEM
Pb	67.42	70.47(+/−0.07)
Ti	14.02	12.06 (+/−0.04)
Pd	3.46	3.45 (+/−0.05)
O	15.10	14.02

**Table 2:** Observed % Pd in nominal 30% Pd-substituted PTO sample.

30% Pd substituted PbTiO_3_ Pb(Ti_0.7_Pd_0.3_)O_2.7_	Calculated wt %	Observed wt % from SEM
Pb	65.60	72.15(+/−0.06)
Ti	16.61	8.35 (+/−0.02)
Pd	10.11	7.77 (+/−0.04)
O	13.68	11.72 (+/−0.06)

### Atomic Force Microscopy (PFM and MFM)

**Figure 3:**
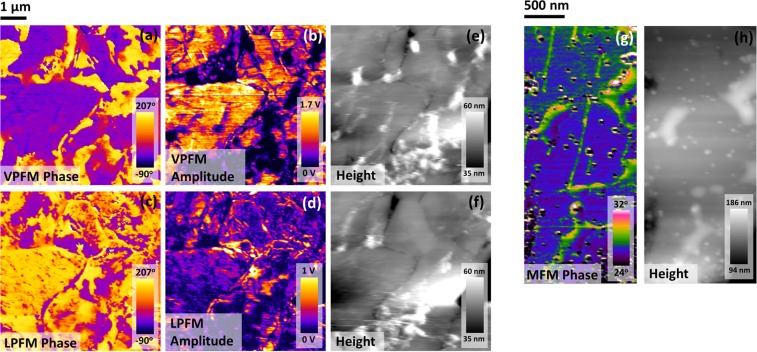
(**a**,**b**) Are vertical PFM micrographs depicting phase and amplitude. Micrographs (**c**,**d**) display lateral PFM phase and amplitude for the same region, as demonstrated by the associated topography maps, shown in (**e**,**f**). (**g**,**h**) Display MFM phase and topography maps. Vertical green streaks, visible in MFM phase but not topography, suggest the existence of magnetic phases that are unlikely to arise due to topographic crosstalk.

In addition to the SEM, to demonstrate the ferroelectric properties of these specimens independent from hysteretic switching, we show in Fig. 3 the piezo-force microscopy (PFM) of domains and domain walls. Note the threefold vertices (bottom left panel) and fourfold vertices (bottom right), in agreement with the model of Srolovitz and Scott^26^. This 4-state planar Potts model is often termed the Ashkin-Teller model after the scientists who first published an equivalent interaction model^27^. It is still studied in the case of an applied external field^28^, which is the case here.

The atomic force microscopy (AFM) in the piezoresponse force microscopy (PFM) images reveal some small amount of exosolution structures on the surfaces of our PZT:Pd^29,30^. This is not unexpected. When studied as catalysts, palladium-doped perovskites usually support elemental Pd and PdO as nano-nodules on their surfaces. Thus one aim of the present study was to determine how much Pd is supported in this way and how much is truly substitutional for Pb or Ti; we find that the Pd is almost fully substitutional (typically > 90%).

The magnetic force microscopy (MFM) used here is sensitive to only out of plane magnetization; therefore systems with magnetic in-plane domains show up in MFM only as walls or Bloch lines between planar domains, since they contain components of M perpendicular to the walls^31^. They are typically very narrow (<25 nm) and exhibit only threefold vertices^32^. This description is satisfied by Fig. 3b below, but the thin green lines cannot be magnetic Bloch lines or walls because they do not move under +0.8 to −0.8 T. Their origin is unknown but suspected to be exsolved lamellar Pd-rich regions in cross-section.

#### Exsolved Pd surface structures

**Figure 4:**
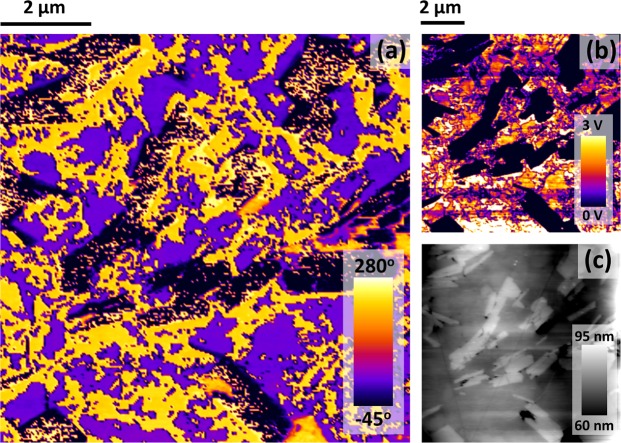
(**a**–**c**) Are micrographs representing vertical PFM phase, amplitude and topographic height respectively. The platelet features, which are seen in the topography map to sit above the ceramic surface, exhibit negligible PFM response, as evidenced through the lack of PFM amplitude signal in (**b**). These platelets are therefore believed to be non-polar.

The AFM studies also reveal exsolved Pd supported at the grain surfaces (evident in both MFM and in topography) in some samples, shown in Fig. 4.

#### Extension of Srolovitz-Scott Vertex Model

In the domain vertex model of Srolovitz and Scott either fourfold domains are unstable and rapidly separate into closely spaced pairs of Y-shaped threefold vertices, or the pairs of threefold domains are unstable and rapidly coalesce into a single fourfold X-shaped vertex; this depends upon parameters in the Potts model used. To be specific, a four-state Potts model is required to describe the domain wall vertices in any case. However, to explain pairs of adjacent three-fold vertices, the Potts model is a scalar model (a simple extension of an Ising model); in this model any fourfold vertex quickly decomposes to adjacent pairs. But a vector Potts model (“clock model”) is required to describe the fourfold vertices. In this case, pairs of adjacent threefold vertices quickly coalesce to X-shaped fourfold vertices. Hence a given sample can exhibit threefold or fourfold vertices but not both. The lower panels in Fig. 3 illustrate both cases, however. This does not violate the Srolovitz-Scott model, but it implies that the interaction parameter is anisotropic, with out-of-plane polarized domains satisfying a scalar n = 4 Potts model and in-plane polarized domains satisfying a clock model. This has not been reported before in any ferroelectric, with the exception of lead strontium titanate (PST)^33^, where pairs of threefold vertices are found and – “very rarely” – a few fourfold vertices (it is tempting to suggest that these very few may have been interrupted in a transient state after nucleation). SrBi_2_Ta_2_O_9_ (SBT) also exhibits both threefold and fourfold vertex geometries^34^. This may give some insight into the more complex circular domains reported recently^35,36^. Note in particular that applying an external electric field to rotate the polarizations will necessarily create and destroy vertex structures, which had not previously been known.

We can suggest a simple physical reason for the out-of-plane domains satisfying a scalar Potts model but in-plane conforming to the vector Potts prediction. In this respect we can point out only that these domains are not topologically equivalent, with out-of-plane polarizations allowed to be only up or down. The collapse of fourfold vertices into adjacent pairs of threefold vertices is further considered by Opperman^37^, who emphasizes qualitative changes in dynamics due to axial anisotropy^38^.

We note parenthetically that n = 4 Potts systems cannot produce Brerezinskii-Kosterlitz-Thouless melting in two-dimensions (although n = 6 can); the n = 4 systems do not form hexatic two-dimensional states^39,40^. It is also worth noting that the two-dimensional Potts model gives second-order phase transitions for n < 4 but first-order for n = 4 or greater, so the n = 4 state is special.

#### Voronoi partitions and n-fold vertices

It is also useful to point out that the general question in nature of whether fourfold or threefold vertices are stable is rather well known. In two dimensions Voronoi partitions (domain walls in the present context) subdivide space into four quarters^39–41^ only when the objects of interest (nucleation sites at defects in the present examples) are symmetrically placed on the vertices of a rectangle. Otherwise, any other configuration generates closely spaced pairs of threefold vertices. This is a result of the growth of domains from initial nucleation sites to fill two-dimensional Euclidean space. Weaire and Aste describe this^39–41^ as a two-dimensional law: “Only three states meet in a common vertex,” or “vertex connectivity =3”. However, in three dimensions the opposite is true, and fourfold vertices are stable; in work to be reported elsewhere we find 100% fourfold vertices in ultra-tetragonal PbTiO_3_ on PbO. Therefore the change of vertex geometry from fourfold for in-plane PZT to threefold for out-of-plane polarization may derive from the effective dimensionality in the two cases: Are our thin films truly two-dimensional? The observation that these rules hold in general for Voronoi partitions implies that the phenomenon may be independent of the nature of interaction (“jamming”) between or among ferroelectric domains. Voronoi partitions are sometimes referred to as Dirichlet tessellations.

Parenthetically, since the number of domains intersecting a vertex must be n > 4 for Kosterlitz-Thouless melting to occur in two dimensions, such melting is unlikely for most ferroelectric domain walls.

### High-pressure Synchrotron studies

In the present situation we employ high-pressure X-ray and Raman studies to elucidate the behaviour of Pd-substituted lead titanate^42,43^. Our earlier X-ray studies (Table 3) at atmospheric pressure show a negligible change in lattice constants with Pd-substitution^44^. This is as expected, since Pd^+2^ fits perfectly into the Pb A-site and Pd^+4^ fits exactly into the Ti B-site. However, it disagrees with the theoretical prediction^45^ that Pd-substitution in PbTiO_3_ should reduce the <a> lattice constant from the accepted literature value of 0.391 nm to ca. 0.380 nm, together with a significant increase in <c>. 30% was the observed saturation limit for Pd-doping, above which phase separation occurs.

**Table 3:** Lattice constants (ambient average of St.-Andrews samples) for Pb(1-x)Pd(x)TiO_3._

Sample	a = b (nm)	c (nm)	V(nm^3^)
PbTiO_3_ (XRD)	0.389743	0.414061	0.062896
Pb(Ti_0.9_Pd_0.1_)O_2.9_ (XRD)	0.390121	0.414276	0.063025
Pb(Ti_0.7_Pd_0.3_)O_2.7_ (XRD)	0.390025	0.414393	0.063037
Pb(Ti_0.9_Pd_0.1_)O_2.9_ (Synchrotron)	0.391	0.421	0.0627

The lattice parameters for Pb_(1-x)_Pd_(x)_TiO_3_ obtained from the XRD (ambient) and from the synchrotron is listed in Table 3.

Because Pd^+4^ is a very good size match for Ti^+4^, it is expected that the phase diagram under hydrostatic pressure is nearly the same with and without Pd substitution. The high-pressure data published on pure PbTiO_3_ do not always agree (perhaps due to unintentional uniaxial strain), so this permits some further clarification of published data.

**Figure 5:**
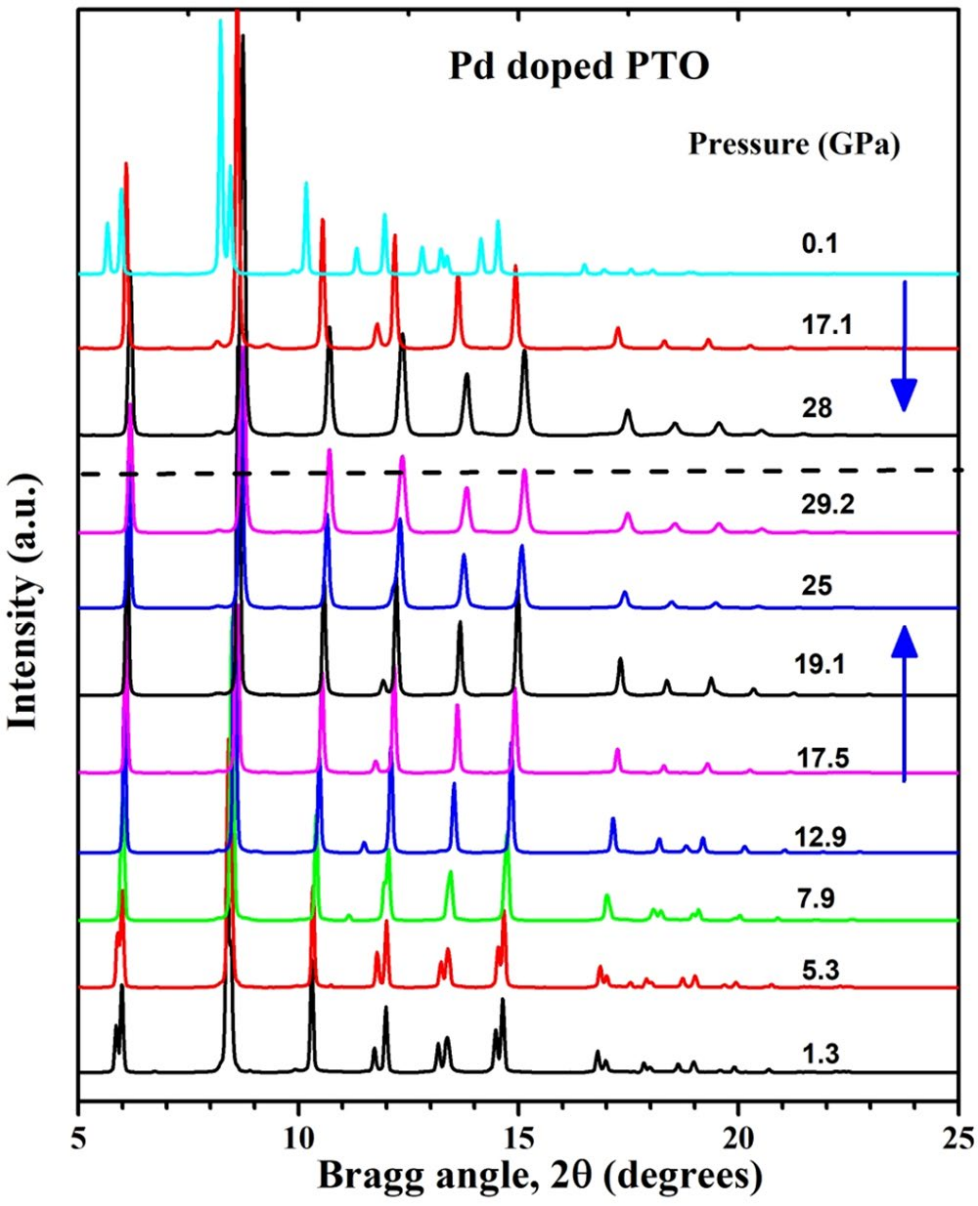
Diffraction pattern of Pb(Ti_0.90_Pd_0.10_)O_3_ stacked at a few representative pressures during compression and release. The diffraction patterns stacked above dashed line are obtained during release of pressure while the one stacked below it are collected during compression.

**Figure 6:**
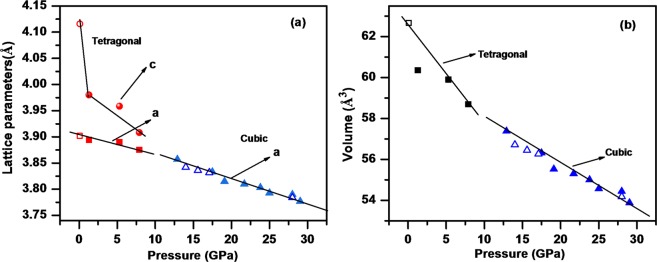
(**a**) <a> and <c> lattice constants for Pb(Ti_0.90_Pd_0.10_)O_3_ versus hydrostatic pressure; (**b**) Unit cell volume V versus pressure. Note that the c-axis lattice constant decreases dramatically in the first 1 or 2 GPa, suggesting a low-P phase transition; this is also observed in pure lead titanate^46^.

Figure 5 shows the diffraction pattern of Pb(Ti_0.90_Pd_0.10_)O_3_ stacked at a different pressures. The diffraction pattern at lower pressures could be fitted to tetragonal phase of PbTiO_3_. This structure persists up to ~7.9 GPa. Beyond this pressure, it transforms to cubic phase^46^ similar to that of the pure PbTiO_3_ which remains stable until 30 GPa, the highest pressure in this study. On complete release of pressure the tetragonal phase appears indicating the reversible nature of this phase transition. Appearance of a new diffraction peak at 2θ = 11.49° (d = 2.03 Å) at 12.9 GPa can be correlated with the possible impurity of palladium oxide. Only one Bragg peak shifts much with hydrostatic pressure (2θ goes from ca. 10.2° to 11.8°), but this line is tentatively assigned to be a second phase (PdO) of a few per cent concentration. In addition, one Bragg peak near 2θ of 13 degrees disappears above 12.5 GPa. This is the pressure at which the high-pressure Raman data below show conversion to cubic Pm3m structure. The observed diffraction patterns have been analyzed using GSAS package to deduce the lattice constant and cell volume data for tetragonal and cubic phases as shown in Fig. 6a,b.

### High-pressure Raman studies

**Figure 7:**
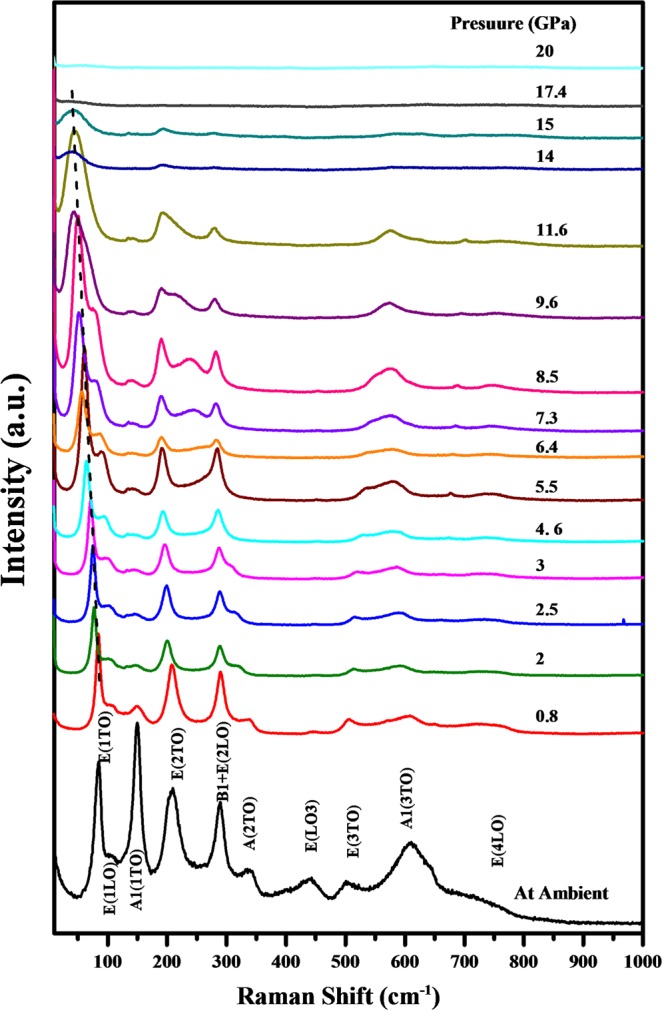
Raman spectra of Pb(Ti_0.90_Pd_0.10_)O_3_ recorded up to 20 GPa. The bottom panel shows the Raman spectra recorded at ambient conditions with the assignment of Raman modes.

**Figure 8:**
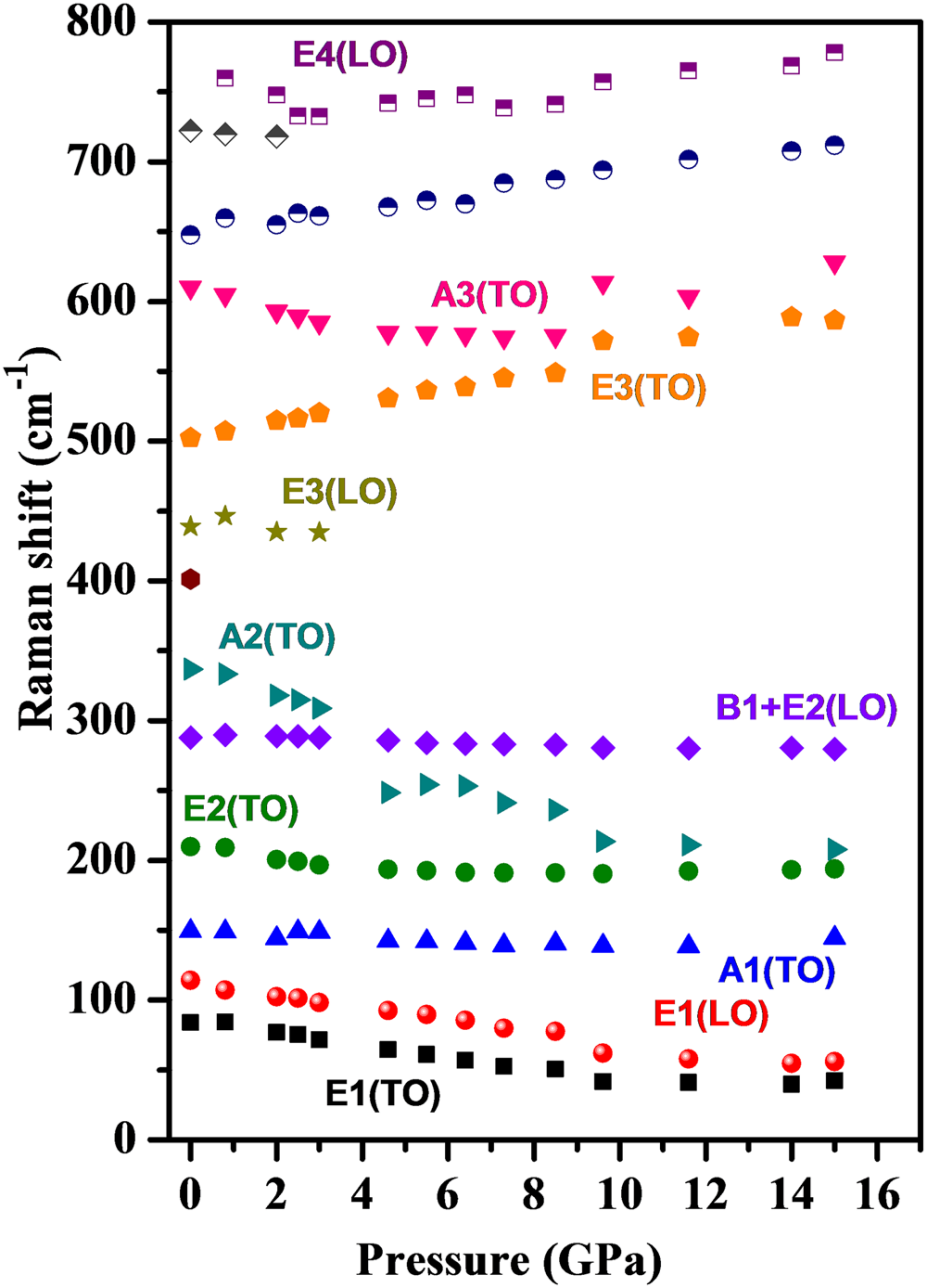
Pressure induced variation of frequencies of Raman modes for Pb(Ti_0.90_Pd_0.10_)O_3_. Their assignments are written along with the modes.

The Raman spectrum of Pb(Ti_0.90_Pd_0.10_)O_3_ recorded at ambient conditions is shown in the lowest panel of Fig. 7. The Raman modes observed in the spectral region 50-1000 cm^−1^ are assigned alongside their mode positions and are in close agreement with the earlier observed Raman modes for perovskite structures^47^. The Raman spectra for Pb(Ti_0.90_Pd_0.10_)O_3_ are fitted and plotted in Fig. 7 at a few representative pressures in the upper panel. The E1(TO) Raman mode observed at ~83 cm^−1^ at ambient conditions softens by ~43 cm^−1^ with pressure until ~9 GPa and beyond this pressure the rate of softening decreases and it remains constant by ~16 GPa. The weak E1(LO) Raman mode observed at ~112 cm^−1^ softens and gains intensity at higher pressures at the cost of E1(TO) Raman mode beyond 7.3 GPa. At further higher pressure the intensity of this mode enhances and it merges into the E1(TO) mode. The intensity of A1(TO) mode observed at ~150 cm^−1^ diminishes suddenly above 0.8 GPa and it becomes discernible beyond 14 GPa pressure. It remains almost invariant with pressure, indicating the rigid nature of this Raman mode. The E2(TO) Raman mode observed at 210 cm^−1^ shows a slight softening until 8 GPa, beyond which it remains almost invariant with pressure, as shown in Fig. 8, whereas the B1 + E2(LO) Raman mode observed at 295 cm^−1^ does not show any significant variation with pressure. On the other hand, the weaker Raman mode A2(TO) observed at 347 cm^−1^ shows drastic softening with pressure and crosses the E2(LO) Raman mode at ~5 GPa. All other higher frequency Raman modes except the A3(TO) Raman mode observed at 608 cm^−1^ show pressure induced stiffening, indicating the increase in corresponding bond strengths, as shown in Fig. 8. Note the soft mode behavior in the middle frame from P = 0.8–4.0 GPa. This suggests a low-pressure phase transition near P = 1 GPa, corresponding to the tricritical line at 1.8 GPa. These spectra are very similar to those in^48^, except that our A_1_/E symmetry mode intensity ratios are much larger; these ratios depend upon grain orientation and laser polarization.

### X-ray Absorption Spectra

**Figure 9:**
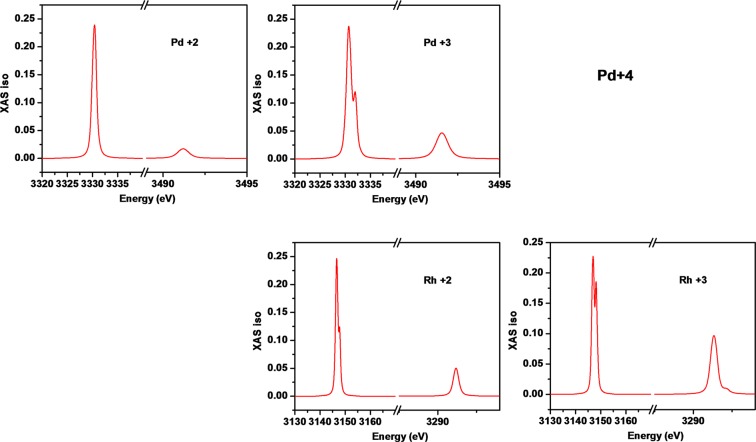
L-edge of Pd in Pb(Ti_0.70_Pd_0.30_)O_3-δ_ sample at 300 K.

**Figure 10:**
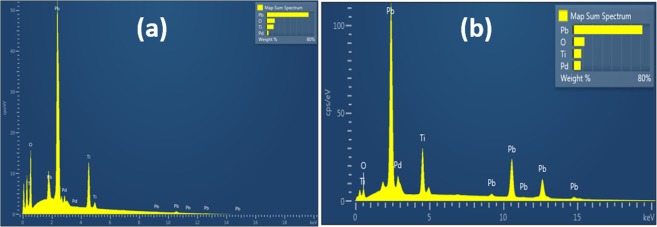
EDS mapping of elements in (**a**) 10% and (**b**) 30% Pd substituted PbTiO_3_.

X-ray absorption spectroscopy (XAS) can provide a very accurate valence over their varying surface probing depths. We have taken advantage of the high flux and energy resolution available at beamline 6.3.1 at the Advanced Light Source at Lawrence Berkeley National Laboratories. To study the valence of Pd, initially we investigated the XAS at the Pd M edge on 30% substituted PTO ceramic, as the L-edge cannot be measured at that beamline’s available energy range; and then we calculated L_3,2_ spectra using the program XClaim, hoping that they are dominated by the final states and thus comparable. The *L*-edge probes the 2p energy levels of elements. The result is shown in Fig. 9, compared with EDS data in Fig. 10. It seems that Pd^2+^ does not really have a shoulder on the L_3_ or L_2_ edge, whereas Pd^+3^ has a shoulder on the L_3_ edge, but not on L_2_. According to the periodic table, Pd prefers to be 2+ or 4+. Pd^4+^ has a shoulder at both L_3_ and L_2_. We approximated Pd4+ through Rh3+, because the program only offers 2+ and 3+. So our hypothesis is that Pd is mostly in the 4+ or 3+ state in this material. We can exclude that much Pd is metallic, because for that the peak splitting is too pronounced. Since we know that metallic Pd and Pd^+3^ are both very unlikely in this material, we conclude that the Pd is mostly 4+. These XAS studies were not extensive and were intended as an independent double-check on the Pd valence.

### Direct Magnetoelectric Measurements

**Figure 11:**
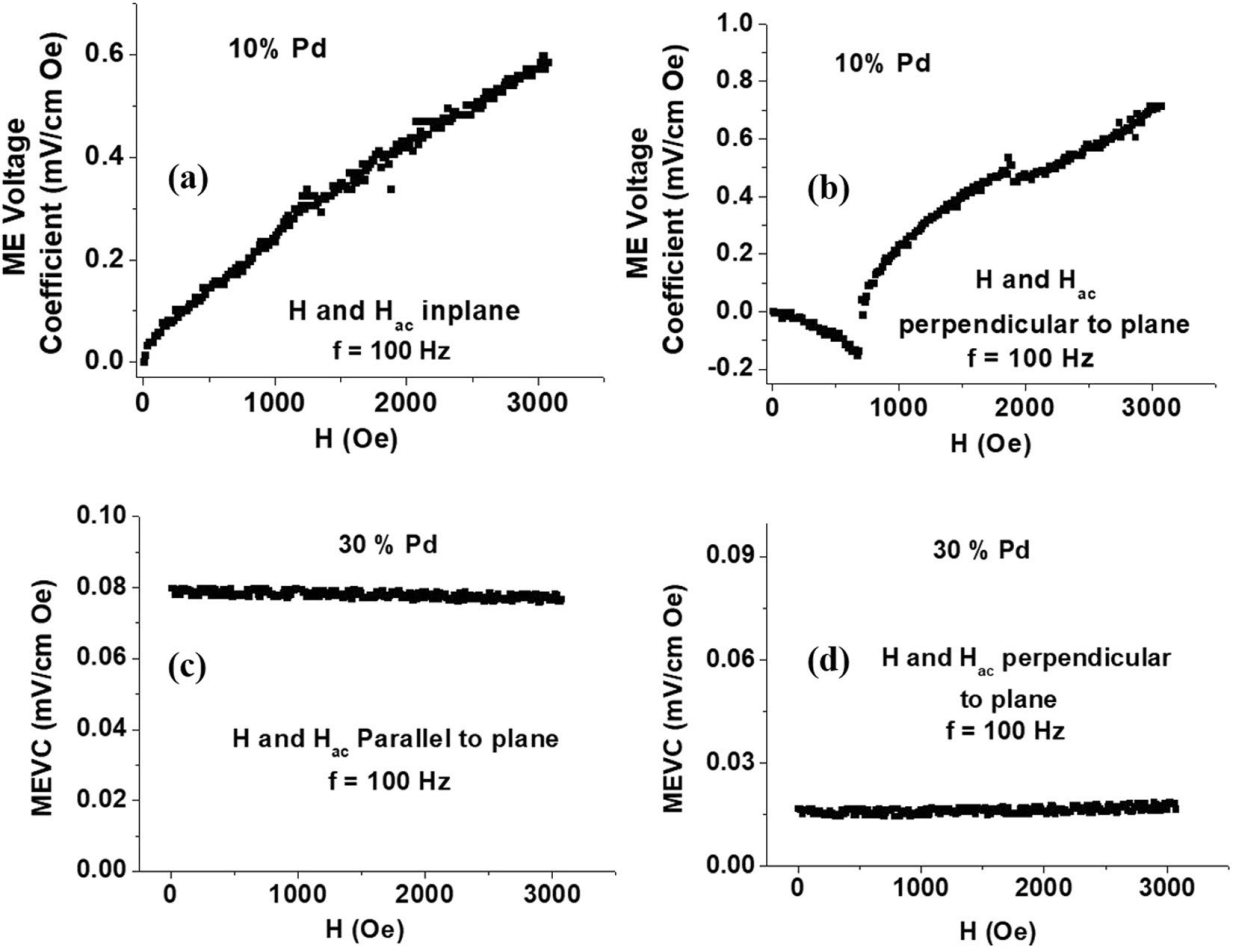
Magnetoelectric measurements at 293K on (**a**,**b**) 10% PbTiO_3_:Pd and (**c**,**d**) PbTiO_3_:Pd with 30% Pd for χ_E,31_.

Figure 11 shows MEVC vs H data for the sample with 10% Pd for magnetic fields either parallel or perpendicular to the sample plane. Results in Fig. 11(a) for in-plane fields show a linear increase in MEVC with H up to a maximum of 0.6 mV/cm Oe for H = 3 kOe. For fields perpendicular to sample plane, the MEVC is negative (a phase difference of 180 deg. with respect to the applied Hac) for H < 1 kOe and it becomes positive and increases with H up to a maximum of 0.75 mV/cm Oe for H = 3 kOe. The ME voltage coefficient is directly proportional to the product of the piezomagnetic coefficient q = dλ/dH (where λ is the magnetostriction) and the piezoelectric coefficient d. For in-plane magnetic fields, assuming sample plane to be (x, y) and H along the x-axis, q_//_ = q_xx_ + q_xy_ In general q_xy_ ~ −0.5 q_xx_, so that q_//_ = 0.5 q_xx_. For out-of-plane fields q_⊥ = _q_zz_ and therefore one expects the MEVC for perpendicular fields to be a factor of 2 higher than the ME response for in-plane fields. Thus the data in Fig. 11 is in qualitative agreement with expected ME response that arises from strain mediated coupling between the ferromagnetic and ferroelectric subsystems.

Figure 11(c,d) shows similar MEVC vs H data for the sample with 30% Pd and the overall ME response is quite small compared to the sample with 10% Pd. As mentioned earlier, there was a significantly large leakage current in the sample that led to loss of magnetic field induced polarization and a reduction in MEVC.

The data in Fig. 11 show a strong ME coupling in the 10%Pd-PTO system compared to Pd-substituted PZT of the composition Pb_0.8_ Zr_0.2_TiO_3._ MEVC of 0.3-0.4 mV/cm Oe were measured at H = 3 kOe for 30% Pd-PZT and are a factor of 2 or more smaller than for the data for 10% Pd in PTO.

## Conclusions

High-pressure synchrotron and Raman data on Pb_(0.9)_Pd_(0.1)_TiO_3_ imply a single chemical perovskite phase with two or possibly three subtle structural transitions under high pressure (the lowest is a tricritical line; the second is to a non-cubic but metrically cubic phase; and the highest is to a true cubic perovskite). The lower one is uncharacterized and occurs near 1 GPa; here there is a very rapid change in lattice constant <c> at P = 0.8 – 2.0 GPa, signalling the tricritical line at 1.8 GPa and ambient temperature. The middle one has c = a and occurs near 10 GPa, but is probably to a monoclinic M_c_ structure, not a simple cubic perovskite (although metrically cubic), since strong Raman lines persist; the upper one is cubic perovskite and occurs near 17 GPa, where all first-order Raman lines disappear (all ions are at inversion sites and hence all phonon branches of odd parity).

The atomic force microscopy (PFM) data reveal the size and threefold (out-of-plane polarization) and fourfold (in-plane) vertex geometries for ferroelectric domains as well as some information on ferroelastic domain walls. This vertex anisotropy is unique, with the exception of a few examples of fourfold vertices in PST and SBT, described by their observers as “rare”.

These materials have potential use as room-temperature multiferroics and also for catalysis^49,50^. We note, however, that other researchers have found it almost impossible to substitute Pd into perovskites such as lead magnesium niobate-tantalate (PMN-PT)^51^; although Pd substitution is important for multiferroic applications, having the Pd exsolve to the surface rather than occupying the B-sites of the lattice is highly favorable for catalysis^18^. It is unlikely that much of the Pd exsolves to the surfaces of our samples, because that would produce PdO or metallic Pd, neither of which is magnetic at room temperature. It has been reported that oxidation/reduction cycles readily transport the Pd ions from substitutional Pd^+4^ at the B-site in perovskites to metallic Pd at the surfaces^52–54^, but even this view is contentious, with Zenou *et al*.^55^. Concluding “… it is only with an extended set of measurements and careful analysis of the x-ray data that one can determine to what extent Pd is actually substituted into the perovskite”. This view is also supported by refs^56,57^ which find that the ratio of surface Pd° to substitutional Pd^+2^ in perovskites depends strongly on the amount of Pd-loading (higher loading increases Pd^0^/Pd^+2^ ratio) and that SnPdO_3_ with Pd^+4^ is a good catalyst for hydrazine. A broader discussion of multiferroic variants of PbTiO_3_ has been given very recently^58^. Our results show that most of the Pd is substitutional; however, atomic force microscopy (PFM) reveals some Pd surface structures.

## Data Availability

Raw data are available to readers by contacting Prof. Scott.
